# The Impact of Foods, Nutrients, or Dietary Patterns on Telomere Length in Childhood and Adolescence: A Systematic Review

**DOI:** 10.3390/nu14193885

**Published:** 2022-09-20

**Authors:** Desirée Valera-Gran, Daniel Prieto-Botella, Miriam Hurtado-Pomares, Eduard Baladia, Fanny Petermann-Rocha, Alicia Sánchez-Pérez, Eva-María Navarrete-Muñoz

**Affiliations:** 1 Grupo de Investigación en Terapia Ocupacional (InTeO), Department of Surgery and Pathology, Miguel Hernández University, 03550 Alicante, Spain; 2 Centro de Análisis de la Evidencia Científica, Academia Española de Nutrición y Dietética, 08007 Barcelona, Spain; 3 Centro de Investigación Biomédica, Facultad de Medicina, Universidad Diego Portales, Santiago 8370109, Chile; 4 Alicante Institute for Health and Biomedical Research (ISABIAL-FISABIO Foundation), 03010 Alicante, Spain

**Keywords:** foods, nutrients, dietary patterns, antioxidant capacity, telomere dynamics, children, adolescents

## Abstract

Environmental factors such as diet can affect telomere length (TL) dynamics. However, the role that children’s and adolescents’ diets play in maintaining TL is not well understood. Thus, we conducted a systematic review to examine the association between the intake of nutrients, foods, food groups, and/or dietary patterns and TL in childhood and adolescence. Following the PRISMA guidelines, we searched MEDLINE via PubMed, Embase, and Cochrane databases and additional registers and methods. The five selected studies were cross-sectional and conducted in children and adolescents aged 2 to 18 years. The main results suggest that a higher consumption of fish, nuts and seeds, fruits and vegetables, green leafy and cruciferous vegetables, olives, legumes, polyunsaturated fatty acids, and an antioxidant-rich diet might positively affect TL. On the contrary, a higher intake of dairy products, simple sugar, sugar-sweetened beverages, cereals, especially white bread, and a diet high in glycaemic load were factors associated with TL shortening. To our knowledge, this is the first systematic review examining the impact of dietary intake factors on TL in childhood and adolescence. Although limited, these results are consistent with previous studies in different adult populations. Further research is needed to ascertain potential nutritional determinants of TL in childhood and adolescence.

## 1. Introduction

Telomeres are conserved tandem nucleotide sequences at the ends of chromosomes, forming a natural protective structure against genome degradation [1]. As a part of the cellular lifetime, this protective function is progressively lost in each cell division cycle resulting in a shortening of telomeres due to incomplete DNA replication and telomerase suppression. This phenomenon is known as the “end replication problem” [1,2]. However, many other physiological processes and telomere-related factors can trigger telomere shortening, along with environmental conditions that raise inflammation and oxidative stress [3]. Although biological ageing can be measured using several biomarkers, telomere shortening is considered a primary ageing hallmark because of its crucial role in cellular senescence and organismal ageing [4]. Therefore, telomere length (TL) is widely accepted to be a reliable biomarker of ageing, morbidity, and mortality [5], remaining one of the most used ageing biomarkers in clinical and epidemiological studies [5,6,7]. As such, ageing-related pathological conditions such as cardiovascular diseases [8], type 2 diabetes [9], or chronic kidney disease [10] were associated with a shortened TL.

The research on ageing determinants has revealed that environmental factors such as stress, physical activity, obesity, or diet, affect health, biological longevity, and TL dynamics [11]. As oxidative stress strongly influences TL shortening [3], a considerable part of this literature comprises studies examining the association between TL and a healthy lifestyle as an antioxidant diet [11]. Some recently published systematic reviews showed that some antioxidant nutrients [12], the intake of fruits and vegetables [12], and healthy food patterns such as the Mediterranean diet [12,13] were associated with a longer TL. In addition, observational studies and randomised controlled trials have suggested that the ratio between omega-3 and omega-6 can positively impact telomerase activity and TL [14,15] and that the consumption of nuts and seeds has a protective effect on TL [16,17].

Although epidemiological studies examining TL in adults have shown relevant insights, a growing interest in studying TL at early life stages has emerged in parallel because of its potential causal role in developing negative consequences in later adult life [18,19,20,21,22]. In addition to being a significant determinant of biological ageing, some researchers have suggested that TL in childhood shortens much more rapidly than in adulthood, especially during the first 20 years of life [23,24]. Thus, prenatal and early-life exposure to environmental and lifestyle factors become a critical window for health outcomes in later life. In this sense, a recent systematic review suggests that the maternal diet consumed during pregnancy could be associated with modulating offspring’s TL [25]. However, to our knowledge, no comprehensive systematic review has assessed the effect of dietary intake in childhood and adolescence on TL.

Given the relevant links between diet and TL dynamics, it is plausible that children’s and adolescents’ nutrition could play a key role in their TL. Under this premise, this study attempts to answer the following research question: Does a high intake of nutrients, foods, food groups, or dietary patterns influence telomere length in children aged 2–18 years more than a low intake? We adopted the working hypothesis that the intake of certain nutrients, foods, food groups, or dietary patterns is associated with different telomere lengths. Therefore, in this systematic review, we aimed to examine the association between intake of nutrients, foods, food groups, or dietary patterns and TL in childhood and adolescence.

## 2. Materials and Methods

### 2.1. Protocol and Registration

We performed this systematic review methodology using the Preferred Reporting Item for Systematic Review and Meta-analysis (PRISMA 2020) statement [26]. Before starting the review, we prepared a protocol describing the review’s rationale, hypothesis, and planned methods. The protocol of the present systematic review is available in the international database of prospective register systematic reviews (PROSPERO), and the registration number is CRD42021266864 (https://www.crd.york.ac.uk/prospero/display_record.php?ID=CRD42021266864).

### 2.2. Search Strategy

We consulted the following databases for the search and retrieval of all potentially relevant studies: MEDLINE via PubMed (accessed 17 June 2021), Embase (accessed 13 June 2021), and Cochrane CENTRAL (accessed 13 June 2021). In addition, to identifying unpublished and ongoing studies, we searched the US National Institutes of Health Ongoing Trials Register (www.ClinicalTrials.gov) (accessed 14 June 2021) and the World Health Organization (WHO) International Clinical Trials Registry (www.who.int/trialsearch) (accessed 14 June 2021). To obtain a complete picture of all the published literature fulfilling the purpose of this review, we performed separate searches for each database by using different combinations of terms. The keywords and search strategies used in each database are available in the Supplemental Material. Finally, we carried out a snowball search strategy by reviewing the list of citations of the studies included after the screening and the list of articles that cite the included study using the Citation Chaser software [27].

### 2.3. Eligibility Criteria

To select the potentially eligible studies, research team members used the PICO (population-intervention–comparator–outcome) criteria for inclusion: “Population” referred to the following: children who were between 2 and 18 years, of both sexes and any ethnicity, and restricted to the healthy population. “Intervention” denoted the following: primary studies or systematic reviews that were assessing nutrients, foods, food, or dietary patterns (the exposure) in childhood and/or adolescence. “Comparator” was not considered as a criterion for selection. “Outcome” referred to the following: children’s or adolescents’ TL measured by any method. There were no limitations concerning the publication status of the research (pre-print, post-print, first online, or final), year of publication, or the language of publication.

### 2.4. Study Selection

All retrieved articles were imported into the reference management software Zotero (version 6.0, Corporation for Digital Scholarship, Vienna, VA, USA) and then exported into the web-based tool PICO Portal (https://picoportal.org/, accessed 25 June 2021), where duplicated items were removed before the blind peer review screening. D.V.-G., M.H.-P., D.P.-B., and E-M.N.-M. screened all titles and abstracts. Then, according to the inclusion criteria, the authors identified the articles as “included”, “dubious”, and “excluded”, including the reasons for exclusion. Finally, both the included and dubious articles were selected for full-text reading.

If there were discrepancies in the inclusion/exclusion process (i.e., screening and identification), E.B. acted as a third reviewer to make a final choice. Otherwise, the group helped in making decisions by consensus, if necessary.

### 2.5. Data Extraction and Synthesis

Data extraction was also performed through a blind peer process. The authors extracted data from the included articles using a form created for this review and previously piloted. The information extracted from each article was the following: the article’s title; the first author; the publication year; the year when the study was conducted; the sample size; the participants’ sex and age; the details about the intervention or exposure factor; the information on the comparison group; the outcomes; the results (association measure and 95% interval confidence); the study’s conclusions; the conflicts of interest; and the funding.

The search and selection process results were summarised using a flow diagram according to the PRISMA 2020 statement [26]. The qualitative synthesis of the characteristics and findings of the studies included is presented in the tables, in line with the methods proposed by the Centre for Reviews and Dissemination [28].

### 2.6. Risk of Bias Assessment

All articles selected for inclusion were cross-sectional studies. E.B. and E.M.N.-M. made a blinded assessment of each study by applying the Joanna Briggs Institute (JBI) (Faculty of Health and Medical Sciences, The University of Adelaide, Adelaide, Australia) tool for appraising analytical cross-sectional studies for use in systematic reviews (available at: https://jbi.global/sites/default/files/2019-05/JBI_Critical_Appraisal-Checklist_for_Analytical_Cross_Sectional_Studies2017_0.pdf, accessed 8 September 2021). This tool consists of a checklist with eight criteria that can be scored as being “yes” (i.e., met), “not” (i.e., not met), “unclear”, or “not applicable”. The JBI Analytical Cross-Sectional Studies Critical Appraisal Tool assesses the study’s methodological quality and covers key aspects of the risk of bias in cross-sectional studies: (1) the inclusion/exclusion criteria of the study participants; (2) the study population and setting; (3) the validity of exposure measurement; (4) the standard criteria used for measurement of the participant condition; (5) the confounding factors; (6) the strategies to deal with confounding factors; (7) the outcome assessment validity; (8) the methods of statistical analysis. D.V.-G. assisted in the assessments when there was a lack of consensus.

## 3. Results

### 3.1. Retrieval and Selection Process of Studies

**Figure 1:**
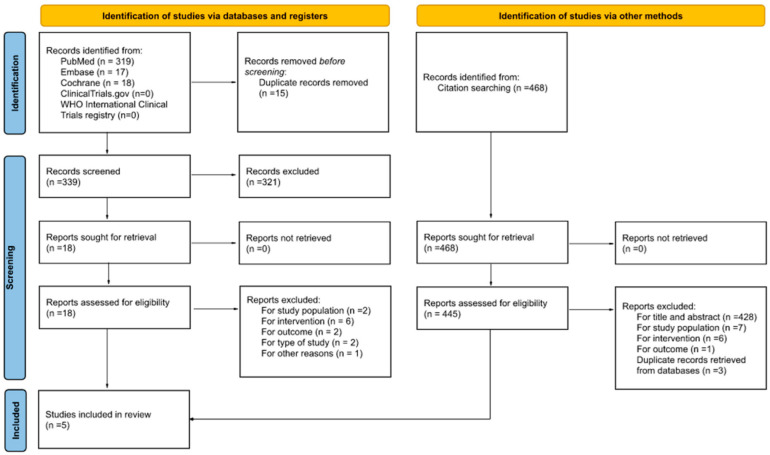
A flow diagram of the search and screening process for identifying the studies.

Figure 1 displays the flow diagram of the search and screening process. First, we identified 354 records from three databases, of which 319 were from Medline via PubMed, 17 from Embase, and 18 from Cochrane. Then, after removing duplicate records, 339 were screened by the title and the abstract. In this part of the process, 321 records were excluded, 137 for not being a part of the target population, 41 for not being intervention/exposure of interest, 2 for not addressing the outcome of interest, 11 for not being the type of study of interest, and 130 for other reasons different from the criteria for inclusion. The next step included 18 articles that were retrieved for a detailed full-text review. Of these, 13 articles were excluded for not meeting the inclusion criteria: 2 for the study population, 6 for the intervention, 2 for the outcome, 2 for the type of study, and 1 for other reasons. Finally, five articles [29,30,31,32,33] were selected for inclusion in this systematic review.

The snowball search yielded 468 additional records for screening, of which 23 were duplicates. After screening for title and abstract, 428 papers were removed, and 17 were selected for a full-text review. Fourteen were excluded for not meeting the study criteria: 7 for the study population, 6 for the intervention, and 1 for the outcome. The remaining three articles were discarded as duplicate records because they were included in the final selection of the articles retrieved from the databases.

### 3.2. The General Characteristics of the Studies Focused on Dietary Determinants of Telomere Length in Children and Adolescents

Table 1 shows the main characteristics and findings of the studies included in this systematic review. All studies had a cross-sectional design, were published between 2015 and 2021, and were conducted on children and adolescents from very culturally different countries. Although children participating in the studies by Baskind et al. [32] and Wojcicki et al. [31] were born in the USA, their mothers were of Latin-American origin. Overall, the study population comprises 1610 individuals (girls = 941, 58.4% and boys = 669, 41.6%), with ages ranging from 2–3 years to 17–18 years.

Regarding dietary intake assessment, two studies evaluated the usual dietary intake using an extensive food frequency questionnaire (FFQ) [29,33]. Todendi et al. examined the consumption of red meat, fish, fruits and vegetables, and fatty foods by adapting several questions from Nahas et al. [34]. In children aged 2–3, Wojcicki et al. [31] only measured the frequency of consumption of sugar-sweetened beverages (SSB) during a month. In girls at 3–5 years of age, Baskind et al. [32] used a specific FFQ to evaluate weekly sugar intake and fast-food consumption. However, apart from using a large 132-item FFQ, the study by García-Calzón et al. [33] made a more detailed assessment of dietary intake by deriving each food item’s nutrient intake, glycaemic load, and the total antioxidant capacity (TAC) from the FFQ. All the studies used the leukocyte TL (LTL) as the outcome, which was calculated as a quantitative real-time polymerase chain reaction (qRT-PCR) based on the method by Cawthon [35,36] and expressed as a relative T/S ratio. For the principal analysis, all the studies used multiple regression models adjusted by potential confounders, among which the child’s age and sex were common co-variates.

**Table 1:** Studies focused on dietary determinants of telomere length in children and adolescents.

Author, Year	Country	Population	Dietary Intake Assessment	Adjustment for Confounders	Main Findings
Meshkani et al., 2021 [29]	Iran	184 children aged 5–7 (girls = 106, boys = 78)	Consumption of foods measured by a FFQ: dairy products, red meat, fish, nuts and seeds, egg, legumes, white bread and refined grains, coloured fruits, other fruits, yellow and orange vegetables, cruciferous vegetables, green leafy vegetables, simple sugar, solid and liquid fats, processed meats, potato chips, carbonated drinks, tea, soft drinks and olives (never or once per month, once per week, and two or three times per week vs. once per day and two or three times per day).	Linear mixed-effect models were fitted with the food group as the fixed effect predictor and PCR plate ID and kindergartens as the random effects Models adjusted by age, sex, BMI, paternal and maternal education level, income, tobacco smoke exposure at home, illiterate per cent per census tract, and unemployed per cent per census tract.	Dairy products and simple sugar were associated with a shorter LTL (β = −0.180; 95% CI: −0.276, −0.085; *p* < 0.001 and β = −0.139; 95% CI: −0.193, −0.086; *p* < 0.001, respectively). Fish (β = 0.208; 95% CI: 0.144, 0.272), nuts and seeds (β = 0.105; 95% CI: 0.041, 0.168), coloured fruits (β = 0.115; 95% CI: 0.047, 0.183), other fruits (β = 0.076; 95% CI: 0.047, 0.183) green leafy vegetables (β = 0.098; 95% CI: 0.037, 0.159), cruciferous vegetables (β = 0.126; 95% CI: 0.067, 0.184), and olives (β = 0.165; 95% CI: 0.108, 0.224) were associated with a longer LTL in children.
Baskind et al., 2021 [32]	USA	97 girls aged 3–5	The weekly consumption of SSB intake (colas/sodas, Kool-Aid, non-diet Hi-C, juices like Capri Sun, Sunny D, and Tampico); fruit juice (100% fruit juice-no added sugar), and flavoured milk (milk flavourings: chocolate, strawberry, etc.); sweets/dessert intake (“cakes, brownies, muffins, donuts, cookies”, “candy or chocolate”, and ice cream consumption. SSB and sweets intake were combined into one sugar intake category. Fast food consumption was also measured as “Fast food: Wendy’s, McDonald’s, Burger King”. Dietary data were categorised into high vs. low intake.	Multivariable models were adjusted by age, maternal education, annual house income, and maternal smoking.	The levels of sugar intake at 3 years were not associated with LTL, although all beta (β) values for the linear regressions were negative. The high SSB intake group, which combined the frequency of consuming soda and soda-like drinks, juice, and flavoured milk (β = −0.07; 95% CI: −0.20, 0.06), was similar to the associations seen in each individual consumption group. The combination category that included both liquid and solid sources of sugar intake, or a “high combined sugar intake”, showed a similar non-significant association (β = −0.08; 95% CI: −0.22, 0.05). A high fast-food consumption greater than once per week did not show any association (β = −0.06; 95% CI: −0.20, 0.08)
Todendi et al., 2020 [30]	Brazil	219 children aged 7–9 (girls = 111, boys = 108) 762 adolescents aged 10–17 (girls = 438, boys = 324)	The frequency of consumption of the following foods based on questions adapted from Nahas et al. [34]: red meat (never or once, 2 to 3 times, or 4 to 5 times a week); fish (never or once, 2 to 3 times, or 4 to 5 times a week); daily diet includes at least 5 servings of fruits and vegetables (never/occasionally or very frequently/always); fatty foods (fats, fried foods), and sweets (never/occasionally or very frequently/always).	Analyses were performed adjusting TL for age, sex, ethnicity, and family income (total sample); and for sex, family income, and ethnicity (separate models for children and adolescents).	Children and adolescents who reported that they always or very frequently ate fruits and vegetables had longer TLs than those who did not (1.17 vs. 1.06, *p* < 0.001). However, when analysed separately, this result was only seen among the adolescents (1.19 vs. 1.04, *p* < 0.001). Although not statistically significant, there was a trend of a longer TL in those individuals who consumed fish four to five times per week or more.
Wojcicki, et al., 2018 [31]	USA	61 children aged 2–3 (girls = 31, boys = 30)	The total number of times that a child consumes SSB (defined as soda, Kool-Aid, Hi-C, sweetened juices, and other beverages with added sugar) over a 1-month period measured continuously.	The model as adjusted by obesity at 6 months and 2–3 years of age, sex, and age of telomere collection at 2–3 years.	Consuming higher levels of SSB was significantly associated with a reduced LTL (β = −0.009; 95% CI: −0.02, −0.0008; *p* = 0.03).
García-Calzón et al., 2015 [33]	Spain	287 participants aged 6–18 (girls = 158, boys = 129)	Food consumption was measured by 132-item FFQ divided into these food groups: dairy products, meat and eggs, fish, fruits and vegetables, legumes, potatoes and cereals, nuts, oils and fat, sweets, and sugar-sweetened beverages. The macronutrients (carbohydrates, protein, and fats [MUFA, PUFA, SFA]) were estimated in %E. The TAC value was calculated in mmol/100 g of food. The glycaemic load for each item was calculated as the total carbohydrate content of each item weighted by its glycaemic index.	The model as adjusted by age, sex, BMI-SDS, and total energy intake (Kcal/d). The dietary TAC and white bread intakes were separately stratified into quintiles and means, and a 95% CI of LTL and were compared in fully adjusted models.	A higher TAC and a greater consumption of PUFA and legumes were associated with longer telomere length (β = 0.173, *p*= 0.007; β = 0.132, *p* = 0.032; β = 0.136, *p* = 0.019, respectively). A higher glycaemic load, cereals, and white bread consumption were associated with shorter telomeres (β = −0.395, *p* = 0.003; β = −0.201, *p* = 0.002; β = −0.204, *p* = 0.002, respectively). Those individuals who had a simultaneously higher dietary TAC (>8.6 mmol) and a lower white bread (<60 g/d) consumption, significantly presented the longest telomeres (β = 0.37, 95% CI: 0.09–0.64). The multivariable-adjusted odds ratio for very short telomeres was 0.30 for dietary TAC (*p* = 0.023) and 1.37 for white bread (*p* = 0.025).

Abbreviations: FFQ, food frequency questionnaire; PCR, polymerase chain reaction; LTL, leukocyte telomere length; BMI, body mass index; CI, confidence interval; TL, telomere length; SSB, sugar-sweetened beverage; MUFA, monounsaturated fatty acids; PUFA, polyunsaturated fatty acids; SFA, Saturated fatty acids; %E, percentage of total energy intake, TAC, total antioxidant capacity; and BMI-SDS, standard deviation score for body mass index.

### 3.3. Quality Assessment of the Included Studies

The results of the quality assessment of the cross-sectional studies included in this systematic review are displayed in Table 2. Using the criteria of the JBI Analytical Cross-Sectional Studies Critical Appraisal Tool, four of the studies met six out of eight, and one did five out of eight. However, the criterion that was used for assessing the quality of the measurement of the participant condition (i.e., A4) was not applicable since the study population consisted of healthy individuals. In addition, all the studies reported unclear information about the validity of the dietary intake measures. Two studies [29,33] reported using a validated FFQ, although the instrument was neither adapted nor validated in the study population. The other three studies reported neither the accuracy nor the validity of the measures that they were based on to assess dietary intake. All studies met the criteria for the remaining aspects of risk of bias, except for the study by Toddendi et al. [30], in which the appropriateness of the statistical analysis (i.e., A8) was unclear.

### 3.4. Dietary Determinants of Telomere Length in Children and Adolescents

The studies included in this systematic review indicated that certain foods, food groups, nutrients, and dietary patterns might be major factors for TL dynamics in childhood and adolescence (Table 1). Regarding foods or food groups, a higher intake of fish (β = 0.208, 95% CI: 0.144, 0.272), nuts and seeds (β = 0.105; 95% CI: 0.041, 0.168), coloured fruits (β = 0.115; 95% CI: 0.047, 0.183), other fruits (β = 0.076; 95% CI: 0.047, 0.183), green leafy vegetables (β = 0.098; 95% CI: 0.037, 0.159), cruciferous vegetables (β = 0.126; 95% CI: 0.067, 0.184), and olives—including olive oil—(β = 0.165, 95% CI: 0.108, 0.224) were associated with a longer LTL in 184 Iranian children aged 5–7 years [29]. Similarly, Todendi et al. showed that the regular intake of fruits and vegetables, compared with no consumption or occasional consumption, may be associated with a longer TL (1.17 vs. 1.06, *p* < 0.001) in 219 Brazilian children aged 7–9 years and in 762 adolescents aged between 10 and 17 years [30]. However, the analysis stratified by groups indicated that this result was only observed among the adolescents (1.19 vs. 1.04, *p* < 0.001). In addition, the study of 287 Spanish participants aged 6–18 years [33] observed an association between greater consumption of legumes and a longer LTL (β = 0.136, *p*= 0.019). On the contrary, the study by Meshkani et al. [29] showed that a higher consumption of dairy products (β = −0.180, 95% CI: −0.276, −0.085; *p* < 0.001) and simple sugar (β = −0.139; 95% CI: −0.193, −0.086; *p* < 0.001) was associated with a shorter LTL. The assessment of SSB in 61 children aged 2–3 years [31] displayed that a higher consumption of these beverages might be associated with a reduction of LTL (β = −0.009; 95% CI: −0.02, −0.0008; *p* = 0.03). However, the assessment of the weekly consumption of sugar and fast food in 97 girls aged 3–5 years showed no association with LTL [32]. García-Calzón et al. [33] showed that a higher intake of cereals (β = −0.201, *p*= 0.002) and white bread (β = −0.204, *p*= 0.002) were factors associated with a shorter LTL. Moreover, in the latter study, multiple logistic regression models disclosed that the consumption of one serving of white bread (i.e., 60 g) per day might be associated with a risk of having the LTL lower than the 10th percentile (OR = 1.37, *p* = 0.025). Regarding specific nutrients or dietary patterns, the only study that examined such nutritional factors was the study by García-Calzón et al. [33]. The results of this study showed that a higher dietary TAC (β = 0.173, *p* = 0.007) and a greater consumption of polyunsaturated fatty acids (PUFA) (β = 0.132, *p* = 0.032) might be positive factors associated with TL. In contrast, the increase in glycaemic load was associated with TL shortening (β = −0.395, *p* = 0.003). Moreover, when analysing TAC and white bread consumption jointly, the results showed that those participants with a higher TAC and who had consumed less white bread showed the longest LTL (β = 0.37, 95% CI: 0.09–0.64). The multiple logistic regression analysis indicated that increasing the intake to six mmol of dietary TAC might protect against having the LTL lower than the 10th percentile (OR = 0.30, *p* = 0.023).

## 4. Discussion

This systematic review examined for the first time the effect of dietary intake on TL in childhood and adolescence. It showed that a higher consumption of fish, nuts and seeds, fruits and vegetables, green leafy vegetables and cruciferous vegetables, olives, and legumes might be associated a with longer TL in middle childhood and adolescence. Moreover, a diet rich in TAC and PUFA was also found to be positively associated with TL at these ages. On the contrary, this review also found that a higher intake of dairy products, simple sugar at 5–7 years of age, as well as higher consumption of cereals, especially white bread, and a diet high in glycaemic load between 6 and 18 years, were factors associated with TL shortening. In addition, this study shows that in toddlers, the intake of higher levels of SSB might also have a negative effect on TL. Although, based on preliminary research, this study has shown that certain foods, nutrients, and dietary patterns may be significant determinants of the biological integrity of TL and that their high or low intake can affect TL dynamics during childhood and adolescence.

According to a recent comprehensive systematic review exploring the impact of nutrition on telomere health in adults [12], the available evidence on the effects of the intake of nutrients, foods, food groups, or dietary patterns is still mainly based on observational research from cross-sectional studies. To date, the results of the effect of fish consumption on TL remain controversial. The positive association between fish and a longer TL reported by Meshkani et al. [29] is consistent with previous cross-sectional studies in the adult population [37,38] and one randomised controlled trial in patients with cardiovascular disease [39]. However, several observational studies with different designs found no association [40,41,42,43,44,45,46,47,48]. A large body of epidemiological research has shown that fish intake has a protective effect on chronic diseases involving systemic inflammation processes [49,50], mainly due to the omega-3 PUFA content of fatty fish. Presumably, it has been hypothesised that the intake of omega-3 PUFA from fish consumption might promote the maintenance of TL through its anti-inflammatory properties. Although the relationship between fish consumption and TL remains unclear, there seems to be evidence that supports the protective action of marine omega-3 PUFA against telomere attrition [14,51,52,53,54,55].

The association between a higher fruit and vegetable intake and a longer TL in Iranian children aged 5–7 years [29] and Brazilian adolescents aged 10–17 years [30] has been supported by some studies conducted in adults [42,43,46,56,57]. On the contrary, the lack of association disclosed by other studies [37,38,41,44,45,48,58,59,60] makes it difficult to determine whether the intake of fruits and vegetables may be beneficial for the stability of TL. Fruit and vegetable intake can supposedly maintain telomeres and limit cell ageing by reducing inflammation and oxidative stress [57]. However, as recognised in previous research, a probable reason for explaining the lack of consistency in the results among studies may be attributed to the different methods used to classify fruits and vegetables as specific foods, groups of foods, and/or eating patterns. Similarly, in line with prior research on the adult population [16,43,61], an association between a higher consumption of nuts and seeds and a longer TL was found in Iranian children aged 5–7 [29]. Furthermore, considering the type of nut, preliminary findings from recent randomised controlled trials have suggested that the consumption of pistachio nuts and walnuts can preserve LTL and reduce oxidative damage to the DNA [17,62]. However, the available evidence on the effects of nuts on the TL has also shown negative or null results [12], suggesting that more research on this issue is required.

Along with the consumption of nuts, the study by Meshkani et al. [29] also showed that olive intake, including olive oil, was positively associated with a longer TL. This finding is consistent with studies that evaluated the association between TL and an adherence to the Mediterranean diet as a proxy measure of olive oil [45,63,64,65]. Furthermore, cumulative evidence supports that dietary olive oil has beneficial compounds against ageing-related diseases and contributes to the maintenance of genomic stability [66]. However, further research evaluating the effects of specifically olive oil intake on TL would be necessary to understand this association properly. In the same way as other food groups, the association between the consumption of legumes and TL remains controversial. For example, the positive effect of a higher intake of legumes observed in children aged 6–18 years [33] is in line with previous research on older ages [37,43], but contradicts the results of other studies [44,45,46,56]. However, among other probable reasons, the findings’ heterogeneity might be likely to be explained by the differences in the dietary assessment due to the wide variety of the foods, by country or culture, that were included in the same food group classification, which discloses the complexity of measuring dietary intake.

Aside from the foods or food groups, other dietary intake factors that were positively associated with TL in the young population found in this review were PUFA and TAC [67]. According to available evidence, the positive effect of PUFA on TL was mainly due to the intake of omega-3 PUFA [14,51,52,53,54,55]. Although PUFA may play a role in regulating gene expression implicated in metabolic alterations and chronic diseases [68], the association between omega-3 PUFA and TL cannot be firmly stated, let alone the influence of omega-6 PUFA or the role of overall PUFA intake concerning TL. Therefore, further research on this nutritional component is needed to clarify its function in telomere biology. From the nutritional epidemiology research perspective, dietary TAC as a global measure of antioxidant status represents a novelty for studying its role in the maintenance of TL. García-Calzón et al. [33] have shown for the first time that dietary TAC is associated with a longer TL in children and adolescents. Based on previous evidence on the Mediterranean dietary pattern [65], the authors inferred that dietary TAC instead of separate food groups might have a stronger influence on TL because of its synergistic effect resulting from the overall interaction among the antioxidants. Interestingly, this dietary pattern-based approach has opened a new line of research on the potential impact of an antioxidant diet on the biological stability of TL.

This systematic review also found that certain foods, food groups, or specific dietary patterns were negatively associated with TL in children and adolescents. In Iranian children aged 5–7 years [29], higher dairy product and simple sugar consumption were associated with TL shortening. Studies evaluating dairy consumption in adults showed inconclusive results, indicating null effects overall [37,40,42,44,47]. However, some studies have reported a positive association [43,46,48]. Lee et al. found a positive association between dairy products and TL in Korean adults aged 40–69 years [43]. Meyer et al. reported that cheese intake positively affected TL, but only in men [48]. Gu et al. observed a positive effect of dairy products in white individuals compared to African American individuals or Hispanics [46]. To our knowledge, only a study with 4029 healthy postmenopausal women has shown a negative association between whole milk or reduced-fat milk and fat-containing cheese [69].

Regarding sugar intake, the study by Meshkani et al. [29] was the first to evaluate simple sugar’s direct effect on TL in children. This finding is in line with the association between higher SSB levels and shorter TL in children aged 2–3 years [31]. Although Basking et al. [32] examined the influence of sugar and fast food intake in children at a similar age, no association with TL was observed. Nevertheless, the negative effect of sugar on TL is consistent with the results of a higher glycaemic load, cereals, and white bread reported by García-Calzón et al. [67] in a sample of 287 Spanish children and adolescents. According to reference values of the glycaemic index, white bread, refined cereals, and sugar are foods with a high glycaemic load [70]. The literature has suggested that consuming foods with a high glycaemic load is associated with increased oxidative stress [71] and inflammatory markers [72]. As in the case of the dietary TAC, the lack of studies examining the effect of a high-glycaemic diet on TL does not allow a direct comparison with the previous studies. However, the glycaemic load as a global measure to detect inflammatory dietary patterns can serve as a helpful tool for investigating the association between the inflammatory status of the diet and TL in the future.

This systematic review is not exempt from limitations. First, the studies included in this systematic review were cross-sectional; thus, significant associations cannot be considered cause–effect relationships. Moreover, the cross-sectional nature of these studies also prevented measuring the inherent differences in TL that were occurring due to the within-person changes over time. Second, the possibility of measurement error in dietary intake cannot be disregarded as the appropriateness of the FFQ for the target population or the validity of the questions used in some studies were not sufficiently specified. Third, although the studies included in this systematic review have taken into account the potential factors related to TL, the presence of residual confounding cannot be dismissed. Finally, although only a few studies have evaluated the potential association between dietary intake factors and TL in childhood and adolescence, the results yielded in most of the studies were consistent with the previous studies with adult populations. However, this systematic review also has several strengths. This study was conducted using clearly defined and reproducible methods to identify and synthesise the results of studies examining the association between the intake of nutrients, foods, food groups, or dietary patterns and TL in childhood and adolescence. This work was conducted in accordance with the recommendations from the PRISMA statement 2020. The study selection, the data extraction, and the assessment of quality were carried out by independent reviewers. In addition, this study sought to determine the dietary factors in childhood and adolescence that support telomere maintenance by promoting or preventing cellular oxidative stress and inflammation. Although the information provided by this systematic review is limited, there are valuable insights into how the dietary intake factors could modulate TL dynamics during childhood and adolescence, which can serve as a basis for future extensive nutritional epidemiological research into TL at the first stages of life.

## 5. Conclusions

The five studies included in this systematic review showed that a higher intake of fish, nuts and seeds, fruits and vegetables, olives, legumes, and PUFA, along with a higher TAC, were associated with a longer TL in children and adolescents. However, a higher intake of dairy products, sugar, cereals, white bread, and a diet with a high glycaemic load were identified as risk factors for TL shortening at these ages. These results are limited but consistent with previous studies conducted in different adult populations, although the information provided from other studies suggests that the evidence in this area remains inconclusive. Therefore, this systematic review highlights the need for further research, mainly from longitudinal studies and randomised controlled trials, to ascertain the potential nutritional determinants of TL in childhood and adolescence.

## Figures and Tables

**Table 2:** Quality assessment of the included studies using the Joanna Briggs Institute Critical Appraisal Checklist for Analytical Cross-Sectional Studies.

	Aspects of Risk of Bias in Cross-Sectional Studies								
Study	A1	A2	A3	A4	A5	A6	A7	A8	Overall Risk of Bias
Meshkani et al., 2021 [29]	yes	yes	unclear	NA	yes	yes	yes	yes	6/8
Basking et al., 2021 [32]	yes	yes	unclear	NA	yes	yes	yes	yes	6/8
Todendi et al., 2020 [30]	yes	yes	unclear	NA	yes	yes	yes	unclear	5/8
Wojcicki, et al., 2018 [31]	yes	yes	unclear	NA	yes	yes	yes	yes	6/8
García-Calzón et al., 2015 [33]	yes	yes	unclear	NA	yes	yes	yes	yes	6/8

Aspects of risk bias: A1, the inclusion/exclusion criteria of the study participants; A2, study population and setting; A3, validity of exposure measurement; A4, standard criteria used for measurement of the participant condition; A5, confounding factors; A6, strategies to deal with confounding factors; A7, outcome assessment validity; and A8, methods of statistical analysis. NA, not applicable.

## Data Availability

Not applicable.

## Supplementary Materials

The following supporting information can be downloaded at: https://www.mdpi.com/article/10.3390/nu14193885/s1, Data S1: Informe de búsqueda (The report on the literature search).

## References

[1] Srinivas N., Rachakonda S., Kumar R. (2020). Telomeres and Telomere Length: A General Overview. Cancers.

[2] O’Sullivan R.J., Karlseder J. (2010). Telomeres: Protecting Chromosomes against Genome Instability. Nat. Rev. Mol. Cell Biol..

[3] Saretzki G. (2018). Telomeres, Telomerase and Ageing. Subcell. Biochem..

[4] McHugh D., Gil J. (2018). Senescence and Aging: Causes, Consequences, and Therapeutic Avenues. J. Cell Biol..

[5] Vaiserman A., Krasnienkov D. (2020). Telomere Length as a Marker of Biological Age: State-of-the-Art, Open Issues, and Future Perspectives. Front. Genet..

[6] Jylhävä J., Pedersen N.L., Hägg S. (2017). Biological Age Predictors. EBioMedicine.

[7] Fasching C.L. (2018). Telomere Length Measurement as a Clinical Biomarker of Aging and Disease. Crit. Rev. Clin. Lab. Sci..

[8] Haycock P.C., Heydon E.E., Kaptoge S., Butterworth A.S., Thompson A., Willeit P. (2014). Leucocyte Telomere Length and Risk of Cardiovascular Disease: Systematic Review and Meta-Analysis. BMJ.

[9] D’Mello M.J.J., Ross S.A., Briel M., Anand S.S., Gerstein H., Paré G. (2015). Association between Shortened Leukocyte Telomere Length and Cardiometabolic Outcomes: Systematic Review and Meta-Analysis. Circ. Cardiovasc. Genet..

[10] Ameh O.I., Okpechi I.G., Dandara C., Kengne A.-P. (2017). Association Between Telomere Length, Chronic Kidney Disease, and Renal Traits: A Systematic Review. OMICS.

[11] Vidacek N.Š., Nanic L., Ravlic S., Sopta M., Geric M., Gajski G., Garaj-Vrhovac V., Rubelj I. (2017). Telomeres, Nutrition, and Longevity: Can We Really Navigate Our Aging?. J. Gerontol. A Biol. Sci. Med. Sci..

[12] Galiè S., Canudas S., Muralidharan J., García-Gavilán J., Bulló M., Salas-Salvadó J. (2020). Impact of Nutrition on Telomere Health: Systematic Review of Observational Cohort Studies and Randomized Clinical Trials. Adv. Nutr..

[13] Canudas S., Becerra-Tomás N., Hernández-Alonso P., Galié S., Leung C., Crous-Bou M., De Vivo I., Gao Y., Gu Y., Meinilä J. (2020). Mediterranean Diet and Telomere Length: A Systematic Review and Meta-Analysis. Adv. Nutr..

[14] Kiecolt-Glaser J.K., Epel E.S., Belury M.A., Andridge R., Lin J., Glaser R., Malarkey W.B., Hwang B.S., Blackburn E. (2013). Omega-3 Fatty Acids, Oxidative Stress, and Leukocyte Telomere Length: A Randomized Controlled Trial. Brain Behav. Immun..

[15] Kalstad A.A., Tveit S., Myhre P.L., Laake K., Opstad T.B., Tveit A., Schmidt E.B., Solheim S., Arnesen H., Seljeflot I. (2019). Leukocyte Telomere Length and Serum Polyunsaturated Fatty Acids, Dietary Habits, Cardiovascular Risk Factors and Features of Myocardial Infarction in Elderly Patients. BMC Geriatr..

[16] Tucker L.A. (2017). Consumption of Nuts and Seeds and Telomere Length in 5,582 Men and Women of the National Health and Nutrition Examination Survey (NHANES). J. Nutr. Health Aging.

[17] Freitas-Simoes T.-M., Cofán M., Blasco M.A., Soberón N., Foronda M., Serra-Mir M., Roth I., Valls-Pedret C., Doménech M., Ponferrada-Ariza E. (2018). Walnut Consumption for Two Years and Leukocyte Telomere Attrition in Mediterranean Elders: Results of a Randomized Controlled Trial. Nutrients.

[18] Factor-Litvak P., Susser E. (2015). The Importance of Early Life Studies of Telomere Attrition. Paediatr. Perinat. Epidemiol..

[19] Blackburn E.H., Epel E.S., Lin J. (2015). Human Telomere Biology: A Contributory and Interactive Factor in Aging, Disease Risks, and Protection. Science.

[20] Blackburn E.H. (2005). Telomeres and Telomerase: Their Mechanisms of Action and the Effects of Altering Their Functions. FEBS Lett..

[21] Aviv A., Shay J.W. (2018). Reflections on Telomere Dynamics and Ageing-Related Diseases in Humans. Philos. Trans. R. Soc. Lond. B Biol. Sci..

[22] Valera-Gran D., Prieto-Botella D., Peral-Gómez P., Hurtado-Pomares M., Sánchez-Pérez A., Navarrete-Muñoz E.-M. (2020). Bibliometric Analysis of Research on Telomere Length in Children: A Review of Scientific Literature. Int. J. Environ. Res. Public Health.

[23] Benetos A., Kark J.D., Susser E., Kimura M., Sinnreich R., Chen W., Steenstrup T., Christensen K., Herbig U., von Bornemann Hjelmborg J. (2013). Tracking and Fixed Ranking of Leukocyte Telomere Length across the Adult Life Course. Aging Cell.

[24] Hjelmborg J.B., Dalgård C., Möller S., Steenstrup T., Kimura M., Christensen K., Kyvik K.O., Aviv A. (2015). The Heritability of Leucocyte Telomere Length Dynamics. J. Med. Genet..

[25] Habibi N., Bianco-Miotto T., Yin Phoi Y., Jankovic-Karasoulos T., Roberts C.T., Grieger J.A. (2021). Maternal Diet and Offspring Telomere Length: A Systematic Review. Nutr. Rev..

[26] Page M.J., McKenzie J.E., Bossuyt P.M., Boutron I., Hoffmann T.C., Mulrow C.D., Shamseer L., Tetzlaff J.M., Akl E.A., Brennan S.E. (2021). The PRISMA 2020 Statement: An Updated Guideline for Reporting Systematic Reviews. BMJ.

[27] Haddaway N.R., Grainger M.J., Gray C.T. (2022). Citationchaser: A Tool for Transparent and Efficient Forward and Backward Citation Chasing in Systematic Searching. Res. Synth. Methods.

[28] Booth A.M., Wright K.E., Outhwaite H. (2010). Centre for Reviews and Dissemination Databases: Value, Content, and Developments. Int. J. Technol. Assess. Health Care.

[29] Meshkani S.E., Kooshki A., Alahabadi A., Najafi M.L., Rad A., Riahimanesh F., Miri M. (2021). Dietary Pattern and Telomere Length in Preschool Children in a Middle-Income Country. Matern. Child Nutr..

[30] Todendi P.F., Martínez J.A., Reuter C.P., Matos W.L., Franke S.I.R., Razquin C., Milagro F.I., Kahl V.F.S., Fiegenbaum M., de MouraValim A.R. (2020). Biochemical Profile, Eating Habits, and Telomere Length among Brazilian Children and Adolescents. Nutrition.

[31] Wojcicki J.M., Medrano R., Lin J., Epel E. (2018). Increased Cellular Aging by 3 Years of Age in Latino, Preschool Children Who Consume More Sugar-Sweetened Beverages: A Pilot Study. Child. Obes..

[32] Baskind M., Hawkins J., Heyman M.B., Wojcicki J.M. (2021). Obesity at Age Six Months Is Associated with Shorter Preschool Leukocyte Telomere Length Independent of Parental Telomere Length. J. Pediatr..

[33] García-Calzón S., Moleres A., Martínez-González M.A., Martínez J.A., Zalba G., Marti A. (2015). GENOI members Dietary Total Antioxidant Capacity Is Associated with Leukocyte Telomere Length in a Children and Adolescent Population. Clin. Nutr..

[34] Nahas M.V., Barros M.V.G., Francalacci V. (2000). O pentáculo do bem-estar: Base conceitual para avaliação do estilo de vida de indivíduos ou grupos. Rev. Bras. Ativ. Fís. Saúde.

[35] Cawthon R.M. (2002). Telomere Measurement by Quantitative PCR. Nucleic Acids Res..

[36] Cawthon R.M. (2009). Telomere Length Measurement by a Novel Monochrome Multiplex Quantitative PCR Method. Nucleic Acids Res..

[37] Zhou M., Zhu L., Cui X., Feng L., Zhao X., He S., Ping F., Li W., Li Y. (2016). Influence of Diet on Leukocyte Telomere Length, Markers of Inflammation and Oxidative Stress in Individuals with Varied Glucose Tolerance: A Chinese Population Study. Nutr. J..

[38] Karimi B., Nabizadeh R., Yunesian M., Mehdipour P., Rastkari N., Aghaie A. (2018). Foods, Dietary Patterns and Occupational Class and Leukocyte Telomere Length in the Male Population. Am. J. Mens. Health.

[39] Corina A., Rangel-Zúñiga O.A., Jiménez-Lucena R., Alcalá-Díaz J.F., Quintana-Navarro G., Yubero-Serrano E.M., López-Moreno J., Delgado-Lista J., Tinahones F., Ordovás J.M. (2019). Low Intake of Vitamin E Accelerates Cellular Aging in Patients With Established Cardiovascular Disease: The CORDIOPREV Study. J. Gerontol. A Biol. Sci. Med. Sci..

[40] Lian F., Wang J., Huang X., Wu Y., Cao Y., Tan X., Xu X., Hong Y., Yang L., Gao X. (2015). Effect of Vegetable Consumption on the Association between Peripheral Leucocyte Telomere Length and Hypertension: A Case–Control Study. BMJ Open.

[41] Bethancourt H.J., Kratz M., Beresford S.A.A., Hayes M.G., Kuzawa C.W., Duazo P.L., Borja J.B., Eisenberg D.T.A. (2017). No Association between Blood Telomere Length and Longitudinally Assessed Diet or Adiposity in a Young Adult Filipino Population. Eur. J. Nutr..

[42] Marcon F., Siniscalchi E., Crebelli R., Saieva C., Sera F., Fortini P., Simonelli V., Palli D. (2012). Diet-Related Telomere Shortening and Chromosome Stability. Mutagenesis.

[43] Lee J.-Y., Jun N.-R., Yoon D., Shin C., Baik I. (2015). Association between Dietary Patterns in the Remote Past and Telomere Length. Eur. J. Clin. Nutr..

[44] Chan R., Woo J., Suen E., Leung J., Tang N. (2010). Chinese Tea Consumption Is Associated with Longer Telomere Length in Elderly Chinese Men. Br. J. Nutr..

[45] Crous-Bou M., Fung T.T., Prescott J., Julin B., Du M., Sun Q., Rexrode K.M., Hu F.B., Vivo I.D. (2014). Mediterranean Diet and Telomere Length in Nurses’ Health Study: Population Based Cohort Study. BMJ.

[46] Gu Y., Honig L.S., Schupf N., Lee J.H., Luchsinger J.A., Stern Y., Scarmeas N. (2015). Mediterranean Diet and Leukocyte Telomere Length in a Multi-Ethnic Elderly Population. Age.

[47] Kasielski M., Eusebio M.-O., Pietruczuk M., Nowak D. (2016). The Relationship between Peripheral Blood Mononuclear Cells Telomere Length and Diet—Unexpected Effect of Red Meat. Nutr. J..

[48] De Meyer T., Bekaert S., De Buyzere M.L., De Bacquer D.D., Langlois M.R., Shivappa N., Hébert J.R., Gillebert T.C., Rietzschel E.R., Huybrechts I. (2018). Leukocyte Telomere Length and Diet in the Apparently Healthy, Middle-Aged Asklepios Population. Sci. Rep..

[49] Jurek J., Owczarek M., Godos J., La Vignera S., Condorelli R.A., Marventano S., Tieri M., Ghelfi F., Titta L., Lafranconi A. (2022). Fish and Human Health: An Umbrella Review of Observational Studies. Int. J. Food Sci. Nutr..

[50] Jayedi A., Shab-Bidar S. (2020). Fish Consumption and the Risk of Chronic Disease: An Umbrella Review of Meta-Analyses of Prospective Cohort Studies. Adv. Nutr..

[51] Da Silva A., Silveira B.K.S., Hermsdorff H.H.M., da Silva W., Bressan J. (2022). Effect of Omega-3 Fatty Acid Supplementation on Telomere Length and Telomerase Activity: A Systematic Review of Clinical Trials. Prostaglandins Leukot. Essent. Fat. Acids.

[52] Fujii R., Yamada H., Munetsuna E., Yamazaki M., Mizuno G., Ando Y., Maeda K., Tsuboi Y., Ohashi K., Ishikawa H. (2021). Dietary Fish and ω-3 Polyunsaturated Fatty Acids Are Associated with Leukocyte ABCA1 DNA Methylation Levels. Nutrition.

[53] Pawełczyk T., Grancow-Grabka M., Trafalska E., Szemraj J., Żurner N., Pawełczyk A. (2018). Telomerase Level Increase Is Related to N-3 Polyunsaturated Fatty Acid Efficacy in First Episode Schizophrenia: Secondary Outcome Analysis of the OFFER Randomized Clinical Trial. Prog. Neuro-Psychopharmacol. Biol. Psychiatry.

[54] Barden A., O’Callaghan N., Burke V., Mas E., Beilin L.J., Fenech M., Irish A.B., Watts G.F., Puddey I.B., Huang R.-C. (2016). N-3 Fatty Acid Supplementation and Leukocyte Telomere Length in Patients with Chronic Kidney Disease. Nutrients.

[55] Farzaneh-Far R., Lin J., Epel E.S., Harris W.S., Blackburn E.H., Whooley M.A. (2010). Association of Marine Omega-3 Fatty Acid Levels With Telomeric Aging in Patients With Coronary Heart Disease. JAMA.

[56] Tiainen A.-M., Männistö S., Blomstedt P.A., Moltchanova E., Perälä M.-M., Kaartinen N.E., Kajantie E., Kananen L., Hovatta I., Eriksson J.G. (2012). Leukocyte Telomere Length and Its Relation to Food and Nutrient Intake in an Elderly Population. Eur. J. Clin. Nutr..

[57] Tucker L.A. (2021). Fruit and Vegetable Intake and Telomere Length in a Random Sample of 5448 U.S. Adults. Nutrients.

[58] Cassidy A., De Vivo I., Liu Y., Han J., Prescott J., Hunter D.J., Rimm E.B. (2010). Associations between Diet, Lifestyle Factors, and Telomere Length in Women. Am. J. Clin. Nutr..

[59] Mirabello L., Huang W.-Y., Wong J.Y.Y., Chatterjee N., Reding D., Crawford E.D., De Vivo I., Hayes R.B., Savage S.A. (2009). The Association between Leukocyte Telomere Length and Cigarette Smoking, Dietary and Physical Variables, and Risk of Prostate Cancer. Aging Cell.

[60] Nettleton J.A., Diez-Roux A., Jenny N.S., Fitzpatrick A.L., Jacobs D.R. (2008). Dietary Patterns, Food Groups, and Telomere Length in the Multi-Ethnic Study of Atherosclerosis (MESA). Am. J. Clin. Nutr..

[61] Zhou J., Zheng Y., Tai J.H.-M. (2020). Grudges and Gratitude: The Social-Affective Impacts of Peer Assessment. Assess. Eval. High. Educ..

[62] Canudas S., Hernández-Alonso P., Galié S., Muralidharan J., Morell-Azanza L., Zalba G., García-Gavilán J., Martí A., Salas-Salvadó J., Bulló M. (2019). Pistachio Consumption Modulates DNA Oxidation and Genes Related to Telomere Maintenance: A Crossover Randomized Clinical Trial. Am. J. Clin. Nutr..

[63] García-Calzón S., Martínez-González M.A., Razquin C., Arós F., Lapetra J., Martínez J.A., Zalba G., Marti A. (2016). Mediterranean Diet and Telomere Length in High Cardiovascular Risk Subjects from the PREDIMED-NAVARRA Study. Clin. Nutr..

[64] García-Calzón S., Martínez-González M.A., Razquin C., Corella D., Salas-Salvadó J., Martínez J.A., Zalba G., Marti A. (2015). Pro12Ala Polymorphism of the PPARγ2 Gene Interacts with a Mediterranean Diet to Prevent Telomere Shortening in the PREDIMED-NAVARRA Randomized Trial. Circ. Cardiovasc. Genet..

[65] Boccardi V., Esposito A., Rizzo M.R., Marfella R., Barbieri M., Paolisso G. (2013). Mediterranean Diet, Telomere Maintenance and Health Status among Elderly. PLoS ONE.

[66] Fernández del Río L., Gutiérrez-Casado E., Varela-López A., Villalba J.M. (2016). Olive Oil and the Hallmarks of Aging. Molecules.

[67] García-Calzón S., Zalba G., Ruiz-Canela M., Shivappa N., Hébert J.R., Martínez J.A., Fitó M., Gómez-Gracia E., Martínez-González M.A., Marti A. (2015). Dietary Inflammatory Index and Telomere Length in Subjects with a High Cardiovascular Disease Risk from the PREDIMED-NAVARRA Study: Cross-Sectional and Longitudinal Analyses over 5 y. Am. J. Clin. Nutr..

[68] González-Becerra K., Ramos-Lopez O., Barrón-Cabrera E., Riezu-Boj J.I., Milagro F.I., Martínez-López E., Martínez J.A. (2019). Fatty Acids, Epigenetic Mechanisms and Chronic Diseases: A Systematic Review. Lipids Health Dis..

[69] Song Y., You N.-C.Y., Song Y., Kang M.K., Hou L., Wallace R., Eaton C.B., Tinker L.F., Liu S. (2013). Intake of Small-to-Medium-Chain Saturated Fatty Acids Is Associated with Peripheral Leukocyte Telomere Length in Postmenopausal Women. J. Nutr..

[70] Atkinson F.S., Brand-Miller J.C., Foster-Powell K., Buyken A.E., Goletzke J. (2021). International Tables of Glycemic Index and Glycemic Load Values 2021: A Systematic Review. Am. J. Clin. Nutr..

[71] Botero D., Ebbeling C.B., Blumberg J.B., Ribaya-Mercado J.D., Creager M.A., Swain J.F., Feldman H.A., Ludwig D.S. (2009). Acute Effects of Dietary Glycemic Index on Antioxidant Capacity in a Nutrient-Controlled Feeding Study. Obesity.

[72] Neuhouser M.L., Schwarz Y., Wang C., Breymeyer K., Coronado G., Wang C.-Y., Noar K., Song X., Lampe J.W. (2012). A Low-Glycemic Load Diet Reduces Serum C-Reactive Protein and Modestly Increases Adiponectin in Overweight and Obese Adults. J. Nutr..

