# Supplementary material for: The Impact of Foods, Nutrients, or Dietary Patterns on Telomere Length in Childhood and Adolescence: A Systematic Review
Source: Nutrients. 2022 Sep 20;14(19):3885. doi: 10.3390/nu14193885 (PMC9570627; doi:10.3390/nu14193885)
Supplement: Supplementary file 1 [file nutrients-14-03885-s001.zip › nutrients-1906133-supplementary.pdf]

INFORME DE BÚSQUEDA

Cliente: Eva María Navarrete Muñoz
Fecha de envío: 21 junio 2021
Referencia: abb-bq001/21

1. Pregunta de investigación.

¿La ingesta alta de nutrientes, alimentos, grupo de alimentos o patrones dietéticos influye en la longitud del telómero en niños de 2 a 18 años más que la ingesta baja?

1.1. Objetivo general

Primarios: Qué asociación existe entre la ingesta de nutrientes, alimentos, grupos de alimentos o patrones alimentarios y la longitud del telómero en la infancia o la adolescencia (2-18 años)
Secundarios:

- Describir las asociaciones entre la ingesta de nutrientes, nutrientes, alimentos, grupo de alimentos o patrones dietéticos y la longitud del telómero según sexos.
- Describir las asociaciones entre la ingesta de nutrientes, nutrientes, alimentos, grupo de alimentos o patrones dietéticos y la longitud del telómero según el método de determinación del longitud del telómero.
- Describir las asociaciones entre la ingesta de nutrientes, nutrientes, alimentos, grupo de alimentos o patrones dietéticos y la longitud del telómero según el tejido donde se realizó la determinación del longitud del telómero.

2. Search Report

Qué asociación existe entre la ingesta de nutrientes, alimentos, grupos de alimentos o patrones alimentarios y la longitud del telómero en la infancia o la adolescencia (2-18 años)
In children 2 to 18 years of age, does high intake of nutrients, foods, food groups, or dietary patterns influence telomere length?

Population	Intervention	Comparator	Outcomes
healthy children 2-to-18 years	nutrients / food / food groups / dietary patterns		telomere length

Type of question	Type of studies
Exposition	Systematic reviews & metaanalysis (1) / CCT (2) / RCT (3) / cohort studies (4) / case-control studies (5) / cross-sectional studies (6) / case reports (7) / practice guidelines (8) / review (9) / cost-analysis (10) / editorial, comments, letter, retracted articles (11)
**Notas**	Gold standard articles: PMID: 25131600 / PMID: 33543592 / PMID: 32558330

Databases	Fecha de búsqueda	Estrategias de búsqueda*	Ítems totales
PubMed	17/06/2021	Appendix 1	319
EMBASE	13/06/2021	Appendix 2	17
CENTRAL	13/06/2021	Appendix 3	18
Clinicaltrials.gov	14/06/2021	Appendix 4	0
IRCTP (WHO)	14/06/2021	Appendix 5	N/A

En los apéndices se incluirá también la historia de la búsqueda en el formato de descarga de la base de datos.

Type of Studies

Bases de datos	1	2	3	4	5	6	7	8	9	10	11
PubMed	5	75	10	55	14	34	13	0	64	2	3
EMBASE	17										
CENTRAL	-	18	-	-	-	-	-	-	-	-	-
ClinicalTrials.gov	-		-	-	-	-	-	-	-	-	-

ICRTP (WHO)	-		-	-	-	-	-	-	-	-	-
**Totales sin duplicados**											

3. Appendix:

3.1. APPENDIX 1. MedLine search strategy via PubMed:

("Telomere"[MeSH Terms] OR "Telomere Shortening"[MeSH Terms] OR "Telomerase"[MeSH Terms] OR "telomer*"[Text Word]) AND ("Diet"[MeSH Terms] OR "diet*"[Text Word] OR "nutrition*"[Text Word] OR "Nutrients"[MeSH Terms] OR "nutrient*"[Text Word] OR "Food"[MeSH Terms] OR "food*"[Text Word] OR "Feeding Behavior"[MeSH Terms] OR "feed*"[Text Word] OR "eat"[Text Word] OR "eating"[Text Word] OR "Alcohol Drinking"[MeSH Terms] OR "alcohol*"[Text Word] OR "drink*"[Text Word]) AND ("Fetus"[MeSH Terms] OR "fetus*"[Text Word] OR "foetus*"[Text Word] OR "fetal*"[Text Word] OR "foetal*"[Text Word] OR "infant"[MeSH Terms] OR "infant, newborn"[MeSH Terms] OR "infant, low birth weight"[MeSH Terms] OR "infant, small for gestational age"[MeSH Terms] OR "infant, very low birth weight"[MeSH Terms] OR "infant, extremely low birth weight"[MeSH Terms] OR "infant, postmature"[MeSH Terms] OR "infant, premature"[MeSH Terms] OR "infant, extremely premature"[MeSH Terms] OR "infan*"[Text Word] OR "newborn*"[Text Word] OR "neonat*"[Text Word] OR "neo nat*"[Text Word] OR "low birth weight*"[Text Word] OR "postmatur*"[Text Word] OR "prematur*"[Text Word] OR "preterm*"[Text Word] OR "Premature Birth"[MeSH Terms] OR "baby"[Text Word] OR "babies"[Text Word] OR "toddler*"[Text Word] OR "Neonatology"[MeSH Terms] OR "Perinatology"[MeSH Terms] OR "perinat*"[Text Word] OR "postnat*"[Text Word] OR "post nat*"[Text Word] OR "child, preschool"[MeSH Terms] OR "schools, nursery"[MeSH Terms] OR "preschool*"[Text Word] OR "pre school*"[Text Word] OR "nursery school*"[Text Word] OR "pre adolescen*"[Text Word] OR "preadolescen*"[Text Word] OR "prepub*"[Text Word] OR "pre pub*"[Text Word] OR "primary education*"[Text Word] OR "primary school*"[Text Word] OR "adolescent"[MeSH Terms] OR "adolescen*"[Text Word] OR "teen*"[Text Word] OR "youth*"[Text Word] OR "juvenil*"[Text Word] OR "Puberty"[MeSH Terms] OR "puber*"[Text Word] OR "pubesc*"[Text Word] OR "child"[MeSH Terms] OR "child*"[Text Word] OR "boy"[Text Word] OR "boys"[Text Word] OR "boyhood*"[Text Word] OR "girl"[Text Word] OR "girls"[Text Word] OR "kid"[Text Word] OR "kids"[Text Word] OR "underage*"[Text Word] OR "under age*"[Text Word] OR "under age*"[Text Word] OR "minors"[Text Word] OR "school*"[Text Word] OR "secondary education*"[Text Word] OR "secondary school*"[Text Word] OR "highschool*"[Text Word] OR "high school*"[Text Word] OR "Pediatrics"[MeSH Terms] OR "pediatr*"[Text Word])

ALTER BIBLIO

3.2. APPENDIX 2. Embase search strategy:

No.	Query Results	Results	Date
#6.	(('telomere'/exp OR 'telomere' OR 'telomere length'/exp OR 'telomere length' OR 'telomerase' OR 'telomere shortening' OR telomer*) AND ('diet'/exp OR 'diet' OR 'nutrition'/exp OR 'nutrition' OR 'nutrient'/exp OR 'nutrient' OR 'food'/exp OR 'food' OR 'feeding'/exp OR 'feeding' OR 'feeding behavior'/exp OR 'feeding behavior' OR food* OR 'eating'/exp OR 'eating' OR 'eating habit'/exp OR 'eating habit' OR eat* OR diet* OR feed* OR nutrition* OR nutrient* OR 'alcohol consumption'/exp OR 'alcohol consumption' OR 'alcohol'/exp OR 'alcohol' OR alcohol* OR 'drinking behavior'/exp OR 'drinking behavior' OR 'drinking'/exp OR 'drinking' OR drink*) AND ([article]/lim OR [article in press]/lim OR [data papers]/lim OR [editorial]/lim OR [erratum]/lim OR [letter]/lim OR [note]/lim OR [review]/lim OR [short survey]/lim) AND ([embryo]/lim OR [fetus]/lim OR [newborn]/lim OR [infant]/lim OR [child]/lim OR [preschool]/lim OR [school]/lim OR [adolescent]/lim)) AND [embase]/lim NOT ([embase]/lim AND [medline]/lim)	17	13 Jun 2021
#5.	('telomere'/exp OR 'telomere' OR 'telomere length'/exp OR 'telomere length' OR 'telomerase' OR 'telomere shortening' OR telomer*) AND ('diet'/exp OR 'diet' OR 'nutrition'/exp OR 'nutrition' OR 'nutrient'/exp OR 'nutrient' OR 'food'/exp OR 'food' OR 'feeding'/exp OR 'feeding' OR 'feeding behavior'/exp OR 'feeding behavior' OR food* OR 'eating'/exp OR 'eating' OR 'eating habit'/exp OR 'eating habit' OR eat* OR diet* OR feed* OR nutrition* OR nutrient* OR 'alcohol consumption'/exp OR 'alcohol consumption' OR 'alcohol'/exp OR 'alcohol' OR alcohol* OR 'drinking behavior'/exp OR 'drinking behavior' OR 'drinking'/exp OR 'drinking' OR drink*) AND ([article]/lim OR [article in press]/lim OR [data papers]/lim OR [editorial]/lim OR [erratum]/lim OR [letter]/lim OR [note]/lim OR [review]/lim OR [short survey]/lim) AND ([embryo]/lim OR [fetus]/lim OR [newborn]/lim OR [infant]/lim OR [child]/lim OR [preschool]/lim OR [school]/lim OR [adolescent]/lim)	320	13 Jun 2021
#4.	('telomere'/exp OR 'telomere' OR 'telomere	4,266	13 Jun 2021

ALTER BIBLIO

	length'/exp OR 'telomere length' OR 'telomerase' OR 'telomere shortening' OR telomer*) AND ('diet'/exp OR 'diet' OR 'nutrition'/exp OR 'nutrition' OR 'nutrient'/exp OR 'nutrient' OR 'food'/exp OR 'food' OR 'feeding'/exp OR 'feeding' OR 'feeding behavior'/exp OR 'feeding behavior' OR food* OR 'eating'/exp OR 'eating' OR 'eating habit'/exp OR 'eating habit' OR eat* OR diet* OR feed* OR nutrition* OR nutrient* OR 'alcohol consumption'/exp OR 'alcohol consumption' OR 'alcohol'/exp OR 'alcohol' OR alcohol* OR 'drinking behavior'/exp OR 'drinking behavior' OR 'drinking'/exp OR 'drinking' OR drink*) AND ([article]/lim OR [article in press]/lim OR [data papers]/lim OR [editorial]/lim OR [erratum]/lim OR [letter]/lim OR [note]/lim OR [review]/lim OR [short survey]/lim)		
#3.	('telomere'/exp OR 'telomere' OR 'telomere length'/exp OR 'telomere length' OR 'telomerase' OR 'telomere shortening' OR telomer*) AND ('diet'/exp OR 'diet' OR 'nutrition'/exp OR 'nutrition' OR 'nutrient'/exp OR 'nutrient' OR 'food'/exp OR 'food' OR 'feeding'/exp OR 'feeding' OR 'feeding behavior'/exp OR 'feeding behavior' OR food* OR 'eating'/exp OR 'eating' OR 'eating habit'/exp OR 'eating habit' OR eat* OR diet* OR feed* OR nutrition* OR nutrient* OR 'alcohol consumption'/exp OR 'alcohol consumption' OR 'alcohol'/exp OR 'alcohol' OR alcohol* OR 'drinking behavior'/exp OR 'drinking behavior' OR 'drinking'/exp OR 'drinking' OR drink*)	5,119	13 Jun 2021
#2.	'diet'/exp OR 'diet' OR 'nutrition'/exp OR 'nutrition' OR 'nutrient'/exp OR 'nutrient' OR 'food'/exp OR 'food' OR 'feeding'/exp OR 'feeding' OR 'feeding behavior'/exp OR 'feeding behavior' OR food* OR 'eating'/exp OR 'eating' OR 'eating habit'/exp OR 'eating habit' OR eat* OR diet* OR feed* OR nutrition* OR nutrient* OR 'alcohol consumption'/exp OR 'alcohol consumption' OR 'alcohol'/exp OR 'alcohol' OR alcohol* OR 'drinking behavior'/exp OR 'drinking behavior' OR 'drinking'/exp OR 'drinking' OR drink*	4,674,784	13 Jun 2021
#1.	'telomere'/exp OR 'telomere' OR 'telomere length'/exp OR 'telomere length' OR 'telomerase' OR 'telomere shortening' OR telomer*	58,022	13 Jun 2021

ALTER BIBLIO

3.3. APPENDIX 3. CENTRAL search strategy:

Date Run: 13/06/2021 18:19:03
ID Search Hits
#1 MeSH descriptor: [Telomere] explode all trees 55
#2 MeSH descriptor: [Telomere Shortening] explode all trees 18
#3 MeSH descriptor: [Telomerase] explode all trees 52
#4 (telomer*):ti,ab,kw (Word variations have been searched) 528
#5 #1 OR #2 OR #3 OR #4 528
#6 MeSH descriptor: [Diet] explode all trees 18928
#7 MeSH descriptor: [Nutrients] explode all trees 5332
#8 MeSH descriptor: [Food] explode all trees 35426
#9 MeSH descriptor: [Feeding Behavior] explode all trees 9101
#10 MeSH descriptor: [Eating] explode all trees 3674
#11 MeSH descriptor: [Alcohol Drinking] explode all trees 3968
#12 (diet* OR nutrient* OR food* OR feed* OR eat* OR alcohol* OR drink*):ti,ab,kw (Word variations have been searched) 190668
#13 #6 OR #7 OR #8 OR #9 OR #10 OR #11 OR #12 198166
#14 #5 AND #13 115
#15 MeSH descriptor: [Infant] explode all trees 32816
#16 MeSH descriptor: [Child] explode all trees 57420
#17 MeSH descriptor: [Adolescent] explode all trees 105819
#18 (child* OR adolescen* OR infant*):ti,ab,kw (Word variations have been searched) 285326
#19 #15 OR #16 OR #17 OR #18 285326
#20 #14 AND #19 18

3.4. APPENDIX 4. ClinicalTrials.gov search strategy:

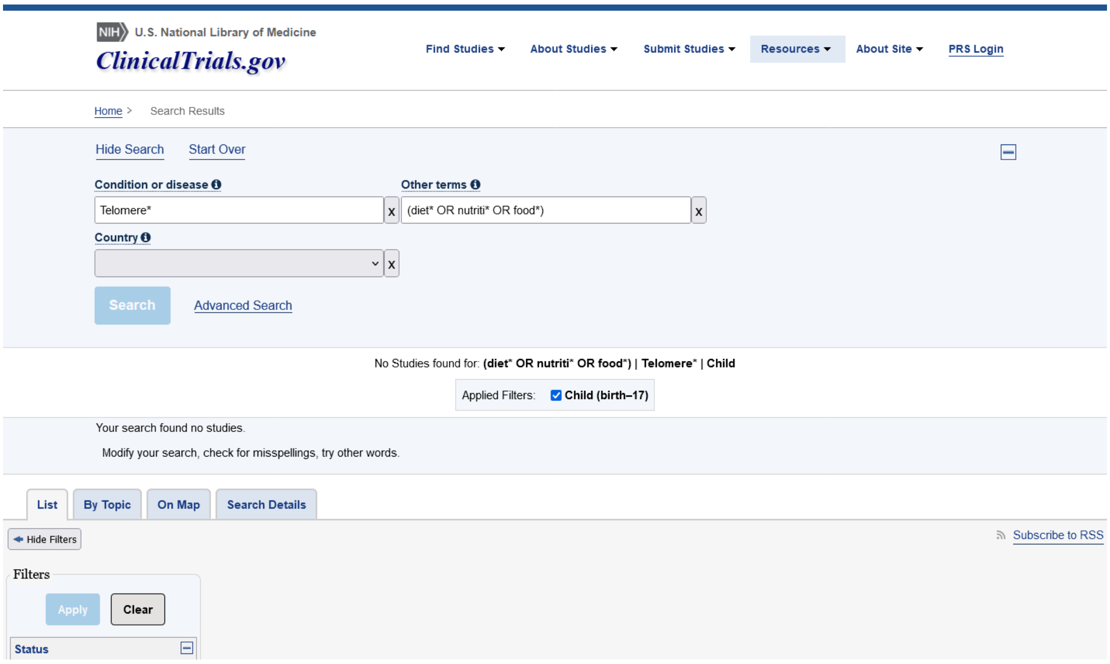

3.5. APPENDIX 5. ICRTP (WHO) search strategy

The requested URL was rejected. Please consult with your administrator.
Your support ID is: 15612090574454605129

ALTER BIBLIO

Contacto

María García-Puente
Alter Biblio
info@alterbiblio.com
(+34) 675143538
-
Referencia de este trabajo:
abb-bq001/21
